# Evaluation of a Viral Microarray Based on Simultaneous Extraction and Amplification of Viral Nucleotide Acid for Detecting Human Herpesviruses and Enteroviruses

**DOI:** 10.1371/journal.pone.0117626

**Published:** 2015-03-16

**Authors:** Yi Liu, Chunhong Duan, Chunxiu Zhang, Xiaomeng Yang, Yan Zhao, Rui Dong, Jiajing Zhou, Zhongtao Gai

**Affiliations:** 1 Pediatric Research Institute, Qilu Children’s Hospital of Shandong University, Ji’nan, China; 2 Department of Pediatrics, Qilu Children’s Hospital of Shandong University, Ji’nan, China; 3 Shanghai Biochip National Engineering Research Center, Shanghai, China; 4 Clinical Central Laboratory, Maternal and Children's Health Care Institute of Jinan, Jinan, China; University of San Francisco, UNITED STATES

## Abstract

In this study, a viral microarray based assay was developed to detect the human herpesviruses and enteroviruses associated with central nervous system infections, including herpes simplex virus type 1, type 2 (HSV1 and HSV2), Epstein-Barr virus (EBV), cytomegalovirus (CMV), enterovirus 71 (EV71), coxsackievirus A 16 (CA16) and B 5(CB5). The DNA polymerase gene of human herpesviruses and 5’-untranslated region of enteroviruses were selected as the targets to design primers and probes. Human herpesviruses DNA and enteroviruses RNA were extracted simultaneously by using a guanidinium thiocyanate acid buffer, and were subsequently amplified through a biotinylated asymmetry multiplex RT-PCR with the specific primer of enteroviruses. In total, 90 blood samples and 49 cerebrospinal fluids samples with suspected systemic or neurological virus infections were investigated. Out of 139 samples, 66 were identified as positive. The specificities of this multiplex RT-PCR microarray assay were over 96% but the sensitivities were various from 100% for HSV1, HSV2, EV71 and CB5, 95.83% for CMV, 80% for EBV to 71.43% for CA16 in comparison with reference standards of TaqMan qPCR/qRT-PCR. The high Kappa values (&gt;0.90) from HSV1, HSV2, CMV, EV71 and CB5 were obtained, indicating almost perfect agreement in term of the 5 viruses detection. But lower Kappa values for EBV (0.63) and CA16 (0.74) displayed a moderate to substantial agreement. This study provides an innovation of simultaneous extraction, amplification, hybridization and detection of DNA viruses and RNA viruses with simplicity and specificity, and demonstrates a potential clinical utility for a variety of viruses’ detection.

## Introduction

Infections of central nervous system (CNS), such as encephalitis, meningitis, and meningoencephalitis, remain a major global cause of morbidity and mortality. The epidemiology of viral infection in CNS has changed dramatically following the introduction of vaccination. Herpes simplex virus type 1 and type 2(HSV1 and HSV2), belonging to genus of human herpes viruses (HHVs), are currently the most common causes of sporadic viral encephalitis; more importantly, the average mortality rate for patients with herpes simplex encephalitis is approximately 70% if left untreated [1,2]. In addition, enteroviruses (EVs) are also the most common causes of viral meningitis [2]. Out of EVs, enterovirus 71 (EV71) and Coxsackievirus 16 (CA16) have often circulated alternatively or together in the Western Pacific region, causing Hand, Foot and Mouth Disease even serious CNS complications in infants and young children in recent years [3,4]. Coxsackievirus B5 (CB5) has been reported to be responsible for recent outbreak in our province [5]. In addition, infections of CNS can also be caused by human cytomegalovirus (CMV) [6,7], Esterin-Barr virus (EBV), and other pathogens [8,9]. Early diagnosis and treatment are crucial to prevent further disease deterioration. However the clinical manifestations of CNS infections from different pathogens are non-specific and clinically indistinguishable, which makes the early diagnosis difficult, it is essential to develop a rapid and comprehensive virology testing technique to diagnose CNS infection and improves the therapeutic management [10,11].

Despite real-time qPCR has been introduced to diagnostic laboratory, only a fraction of pathogens can be detected in patients with suspected CNS infectious syndromes. Nevertheless, there are potential risks of cross-contaminations in qPCR process due to its high sensitivity [12]. The recent advancement of diagnostic for infectious disease relies on development of multiplexing technologies to simultaneously identify more than one pathogen in clinical specimens [13]. Microarray with pathogen-specific probes has become one of the most powerful tools in microbial diagnosis as it enables high throughput assays that in principle capable of identifying every microorganism in a specimen [14]. So far, several microarrays for detection of viruses associated with CNS infections have been reported [15–19]. For example, Korimbocus et al [15] designed a DNA microarray to detect three species of viruses, including HHVs, EVs and flaviviruses, which demonstrated the feasibility of identifying various virus species on one array but showed fatal defects of separate isolation and amplification of different DNA and RNA viruses. Zheng et al. developed a DNA microarray based on multiplex PCR to detect seven HHVs simultaneously in a single specimen [16].

This study aims to evaluate the feasibility of an oligonucleotide microarray based on simultaneous extraction and amplification of DNA and RNA viruses to identify different HHVs and EVs in a single clinical specimen.

## Materials and Methods

### Viruses and cells

Herpes simplex virus type 1 (HSV1; reference stain *stocker;* cultured in Vero cells originated from Cercopithecus aethiops (ATCC,CCL-81)), Herpes simplex virus type 1 (HSV2; reference strain *sav;* cultured in Vero cells originated from Cercopithecus aethiops (ATCC, CCL81)), Epstein-Barr virus (EBV; reference stain *B95-8*, transformed B95-8 cells originated from Saguinus Oedipus (ATCC,VR-1492)), cytomegalovirus (CMV; reference stain *AD169*; propagated in Human embryo lung cells (MRC-5, ATCC CCL-171)), Enterovirus 71 (EV71, clinical isolate from throat swab and confirmed by sequencing; cultivated in Human rhabdomyosarcoma cells (ATCC CCL-136)), Coxsackievirus A 16 (CA16, clinical isolate from throat swab and confirmed by sequencing; cultivated in Human rhabdomyosarcoma cells (ATCC CCL-136)), Coxsackievirus B5 (CB5, clinical isolate from cerebrospinal fluid and confirmed by sequencing; cultivated in Hep-2 cells (ATCC CCL-23)). All viruses were cultured in MEM medium (or RPMI-1640 medium only for B95-8 cells) supplemented with 10% fetal bovine serum at 37°C in Shandong Institute of microbiology, Shandong Academy of Medical Sciences (Jinan, China) until be harvested and then supplied to us.

### Clinical samples

Clinical specimens of 90 blood samples and 49 cerebrospinal fluids (CSFs) samples were obtained from a majority of hospitalized children and a few outpatients with suspected systemic or neurological virus infections at Qilu Children’s Hospital, Shandong Provincial Hospital and Shandong Center for Disease Control and Prevention from October 2012 to June 2013. In addition, 30 blood samples from healthy children and 25 CSF samples from children with clinical symptoms uncorrelated to viral infections were collected for control Samples.

The clinical specimens were tested for HSV1, HSV2, EBV, CMV, EV, EV71 and CA16 by using real-time qPCR/qRT-PCR kits (DAANGENE, Guangdong, China) following the manufacturer’s instructions.

### Ethics statement

The work of using retrospective clinical specimens was approved by Medical Ethics Committee of Qilu Children’s Hospital of Shandong University. The parents of each patient gave their written informed consent before clinical examination was performed. All patients’ information was anonymized and de-identified prior to analysis. The relevant regulations and institutional polices were followed strictly.

### DNA and RNA extraction

DNA and RNA were extracted simultaneously from cell cultures of viruses and clinical samples based on previously described method with slight modification [20]. Briefly, 100 μl of virus culture or clinical samples were mixed with 200 μl of guanidinium thiocyanate acid (GuSCN) lysis buffer (including 4 M GuSCN, 0.5% N-lauroyl sarcosine, 1 mM DTT, 25 mM Sodium Citrate and 0.01% Glycogen.) and incubated for 10 min at room temperature. Then 250 μl of cold isopropyl alcohol was added and centrifuged for 10 min at 12000 rpm at 4°C. After washing using 70% ethanol, 20 μl of diethypyrocarbonate treated water was used to dissolve the pellet.

### Primers and Oligonucleotide Probes

The DNA polymerase gene of HHVs and the 5’-UTR (untranslated region) of EVs were selected as the target genes [15,16]. The primers and probes were designed using an online primer designing tool—Primer 3. Three primers of HHV-f, HHV-r, EBV-r primers and 4 probes for HHVs were finally determined for the detection of HSV1, HSV2, EBV and CMV; two primers of EV-f, EV-r and 3 probes for EVs were used to detect EV71, CA16 and CB5. An oligonecleotids of biotin labeled poly (dT)_15_ and a bacteria universal sequence of 16S rRNA gene were used as the positive and negative conrrol probes, respectively. All primers and probes were synthesized by Shanghai Invitrogen Biotechnology Company (Shanghai, China). The 5’ end of forward primers including HHV-f and EV-f was labeled with biotin (Bio). The sequences of the primers and probes used in this study were shown in Table 1 and Table 2.

**Table 1:** Sequences of the primers used in this study.

Virus		Primer sequences (5′-3′)	Product
**DNA Virus**	**HSV1HSV2 CMV**	HHV-f:Bio-TCATCTACGGGGACACGGAC	232bp
		HHV-r: CGCACCAGATCCACGCCCTT	232bp
	**EBV**	HHV-f:Bio-TCATCTACGGGGACACGGAC	227bp
		EBV-r: GAGCTCCACCCCCTTCATC	
**RNA Virus**	**EV71 CVA16CVB5**	EV-f: Bio-ttaaaacagcctgtgggttg	328bp
		EV-r: tgactcatcgacctgatctaca	

**Table 2:** Sequences of the probes used in this study.

Virus		Probe sequences (5′-3′)
**Positive control**		Biotin-poly(dT)_18_
**Negative control**		TGA CGG GCG GTG TGT ACA AG
**DNA Virus**	**HSV1**	GGCACAGCACAAAGATGGA
	**HSV2**	GGCACAAAACGAAAATGGA
	**EBV**	CGGCACTCGATAAACAGCG
	**CMV**	ACGAAAGCGGACAAACACG
**RNA Virus**	**EV71**	GATCGTGGTTCGCTGCTTCTA
	**CVA16**	CACTATTGGTCGTGATTGTACAAAG
	**CVB5**	TGCCTACTATTGATCGAGGTGTATTG

### Biotinylated asymmetry multiplex-RT-PCR

After extraction of DNA and RNA simultaneously from different viral cultures and clinical samples, transcription reaction (RT) was firstly performed with TaKaRa PrimeScript RT kit (TaKaRa Biotechnology Co, Dalian, China). Briefly, 2 μl of extracted DNA and RNA were added into 10 μl of transcription reaction mixture containing RT buffer (dNTPs and Mg^2+^), 2 μM EV-r (Table 1), and RT Enzyme Mix. The mixture was incubated at 42°C for 15 min and then 85°C for 5 sec. The RT products were utilized as the templates for the next PCR amplification.

The biotinylated asymmetry multiplex-PCR amplification was carried out in single reaction with five primers, including two biotinylated forward primers (HHV-f and EV-f, see Table 1) and three reverse primers (HHV-r, EBV-r and EV-r, see Table 1) that predicted to amplify different HHVs and EVs. In short, a 15μl reaction mixture containing 0.6 μl of sterile distilled water, 0.4 μl of 10 mM dNTPs, 1.5 μl of 10×PCR buffer (100 mM Tris-HCl [pH8.3], 500 mM KCl), 0.6 μl of 15 mM MgCl_2_), 10.4 μl of biotin labeled primers mix (4.5μl of 20 μM Bio-HHV-f, 4.5μl of 20 μM Bio-EV-f, 0.6μl of 20 μM HHV-r, 0.2 μl of 20 μM EBV-r, 0.6μl of 20 μM EV-r), 0.5 μl of Taq DNA polymerase (5U/μl), and 5 μl of the templates. The PCR reaction was performed in a DNA Engine Tetrad Thermal Cycler (MJ Research, San Francisco, CA, USA) and initial denaturation was at 95°C for 5 min; and then 35 cycles at 95°C for 30 sec, 58°C for 30 sec, 72°C for 30 sec; a final extension at 72°C for 5 min. The presence of a PCR product was examined by 2% agarose electrophoresis and visualized with ethidium bromide staining.

### Fabrication of microarray

**Fig 1:**
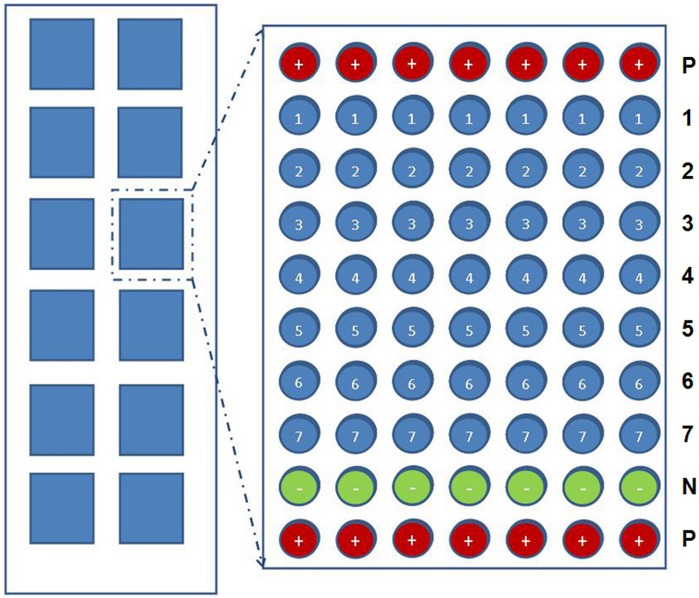
The patterns of viral array. The microarrays were constructed in which oligonucleotides specific probes for 7 indicated viruses were spotted onto glass slides with 7 replicates per row (right). Top and bottom rows contained the positive control consisting of 7 replicates of biotin labeled poly(dT)15. One row contained the negative control of a bacteria universal 16S rRNA gene sequence. The numbers (1–7) on the right of rows represented 7 viral probes. Each array was repeated 12 times per slide (left). P: positive control; N: negative control; 1, HSV1 probe; 2, HSV2 probe; 3. EBV probe; 4. CMV probe; 5. EV71 probe; 6. CA16 probe; 7. CB5 probe.

The nine probes, including a positive control, a negative control and 7 virus-specific oligonucleotides probes described in Table 2, were diluted to 50 μM with DNA spotting buffer (CapticalBio Corporation, Beijing, China) following the manufacture’s instruction. The probes were then added into a 384-well microplate (Corning Life Sciences, Tewksbury, MA, USA) and spotted on aldehyde coated glass slides (Shanghai Biotech Co., Shanghai, China) with a contact microspotting robotic system (OminiGrid 100 Arrayer, GeneMachines, San Carlos, CA, USA). Each slide contained 12 identical arrays on which the nine probes for 7 s viruses, a positive control and a negative control (Table 2) were arranged. Each probe was printed seven replicates dots with 300 μm of spacing and 100 μm of diameter. Each individual line of seven dots from number 1 to 7 represented one specific virus. The positive control probe was spotted on the top and bottom line while the negative control was displayed on the penultimate line. The patterns of the viral array with nine probes were indicated in Fig. 1.The relative humidity and temperature of spotting environment were about 70% and 25°C. The spotted slides were stored in drying cabinet until use.

### Hybridization of microarray and detection of signals

Fifteen microliters of biotin-labeled PCR products were denatured at 95°C for 3 min and immediately cooled on ice for 5 min. Then the denatured PCR products were mixed with equal hybridization buffer (Invitrogen, Shanghai, China.). All the liquid was loaded onto the array slide which was then covered with a clover glass. Hybridization was carried out at 48°C for 2 h in a hybridization oven (UVP HB-1000 Hybridizer, UVP, Upland, CA, USA). After binding Cy3-streptavidin (Invitrogen, Shanghai, China) for 30 min at room temperature, the hybridized array was performed two subsequent washing steps with Wash Buffer A and B (Shanghai Biotech,Co., Shanghai, China) following the manufacture’s instruction. The array slide was dried at room temperature and then fluorescence signals were scanned by Confocal Laser Scanner (Axon GenePix 4000B; GeneMachines, San Carlos, CA, USA) at 100% ScanPower, 500 PMTGain, and 532 nm Wavelengths.

### Generation of recombinant positive controls

To determine specificity and sensitivity of this viral array for detection of 7 viruses, target fragments of 7 viruses were amplified separately with the primers (Table 1) and cloned into pGEM-T vector (Promega, Madison, USA) following the manufacturer’s instructions. Recombinant plasmid DNA were transformed into DH5α competent cells and screened with blue-white selection. The positive plasmids were confirmed by sequencing and then further cultured to generate large quantities and purified DNA with PureYield Plasmid Miniprep kit (Promega, Madison, USA) according to the instruction of the manufacturer. The RNA standards were generated from plasmids with T7 transcription system (Promega, Madison, USA) [21]. The copy numbers of positive controls of viruses were calculated via the absorbance value in combination with Avogadro’s Number (6.022 × 10^23^) as described before [21].

### TaqMan qPCR

TaqMan qPCR kits (DAANGENE, Guangdong, China) were used to test DNA viruses including HSV1, HSV2, EBV and CMV from clinical samples in clinical labs following the manufacturer’s instructions. In brief, the viral DNA was extracted with the reagents provided in the kits from 100 μl of sample suspension with 100 μl of Buffer A. Lysis was then performed at 56°C for 10 with 20 μl of proteinase K and 200 μl Buffer B. All samples were precipitated by using equal volume of absolute ethanol and then centrifuged. The DNA was washed and then eluted as the template for PCR with 20 μl of Buffer C. The qPCR was performed immediately in 20 μl reaction mixture containing 15 μl of amplification mixture and 5 μl of extracted DNA in a qPCR machine (ABI 7500 real-time PCR system, AB Applied Biosystems, Foster City, CA, USA) under the following conditions: denaturation at 94°C for 3 min; then 40 cycles at 94°C for 15 sec and at 55°C for 45 sec.

### TaqMan qRT-PCR

RNA viruses including universal enterovirus (EV), EV71 and CA16 were detected by using TaqMan qRT-PCR kits (DAANGENE, Guangdong, China) following the manufacturer’s instructions. Briefly, the viral RNA of clinical samples was extracted with the reagents provided in the kits from 100 μl of sample mixed with 200 μl of Trizol and 100 μl chloroform. The supernatant was transferred to a new tube after centrifuge at 12,000 rpm for 2 min, and then 10 μl of RNA extraction buffer A was added and centrifuged. The viral RNA was dissolved in 20 μl of DEPC water after washing with 400 μl of the solution C and subsequently were utilized as the template. The qRT-PCR was done by adding 15μl of RT-PCR amplification mixture and 5 μl of RNA template in a qPCR machine (ABI 7500 real-time PCR system, AB Applied Biosystems, Foster City, CA, USA) under the following conditions: one cycle at 45°C for 25 min and at 94°C for 3 min; then 40 cycles at 94°C for 15 sec and at 55°C for 45 sec.

### Conventional RT-PCR and sequencing

For two samples with universal EV positive but EV71 and CA16 negative in qRT-PCR assays, conventional RT-PCR was performed by using PrimeScript RT and PCR kits (TaKaRa Bio, Dalian, China) following manufacturer’s instructions. The reverse transcription (RT) was carried out in a 10 μl of transcription reaction mixture including 2 μl of 5×RT Buffer, 0.5μl of RT Enzyme Mix, 0.5μl of 2μM primer EV-r and 2μl of RNA template. RT was run at 42°C for 15 min and at 85°C for 5 sec. Two microliters of RT products were added to a 23 μl PCR amplification mixture containing 10×PCR Buffer, dNTPs, Taq polymerase, primers EV-f and EV-r. The amplification was run DNA in a DNA Engine Tetrad Thermal Cycler (MJ Research, San Francisco, CA, USA) with denaturation at 94°C for 3 min and then 40 cycles at 94°C for 30 sec, at 58°C for 30 sec and at 72°C for 40 sec; final extension at 72°C for 5 min. The sequencing was performed on genetic analyzer (3730 DNA Analyzer, Life Technologies, Grand Island, NY, USA)

### Data analysis

The quantification of fluorescence intensity and data analysis were conducted by using the Acquisition and Analysis Microarray software (Gene PixPro 6.0, Sunnyvale, CA, USA). The signal-to-noise ratio (SNR) between the average fluorescence intensity from all seven detection spots within each line and the average background intensity was taken for the detection criterion. The threshold of detection was determined as SNR 3.0. Signal values higher than SNR 3.0 were regarded as positive.

The differences in the detection results between the microarray and qPCR/qRT-PCR were analyzed using the Chi square test. The diagnosis sensitivity and specificity, as well as Cohen's Kappa were determined in comparison with qPCR/qRT-PCR and were caculated with conventional formulas.

## Results

### Specificity of multiplex RT-PCR-microarray

**Fig 2:**
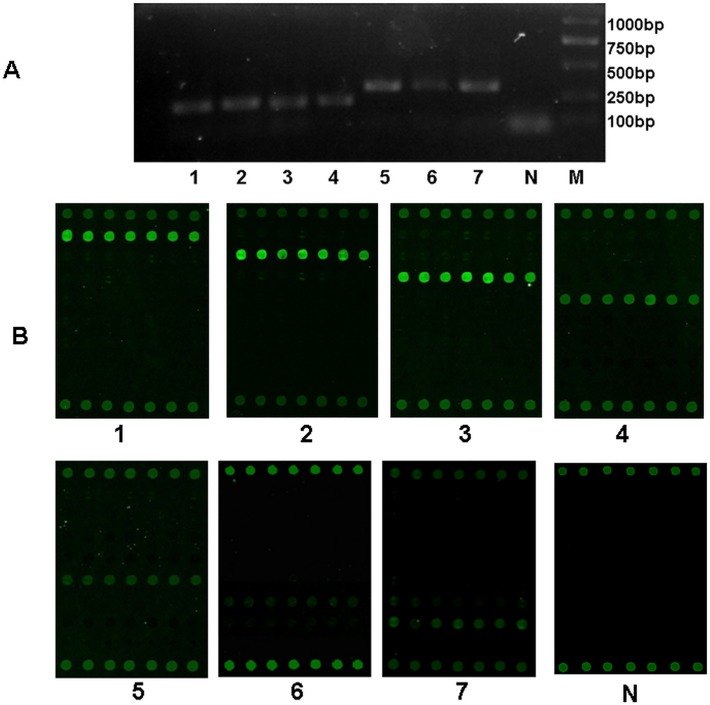
Result of multiplex RT-PCR and microarray for detection of 7 viruses. A. Result of asymmetry multiplex RT-PCR for 7 viruses. Nucleic acids were extracted from simulative samples mixed the recombinant viral controls with a negative CSF sample, and then were amplified by use of multiplex RT-PCR as described in Materials and Methods. PCR products were separated by agaraose gel electrophoresis and visualized by ethidium bromide staining. B. Result of microarray for 7 viruses. Biotin-labeled PCR products (shown in panel A) were hybridized to the microarray and detected using Cy3-labled streptavidin then visualized by confocal laser scanner as described in Material and Methods. The numbers of 1–7 represented the 7 viruses: 1, HSV1; 2, HSV2; 3, EBV; 4, CMV; 5, EV71; 6, CA16; 7, CB5. N, negative control.

To determine the specificity of the multiplex RT-PCR microarray assay, 6 × 10^2^ copies/μl of viral positive controls were prepared by using 7 recombinant viral nucleic acid spiked individually into 7 aliquot of a CSF sample that was negative for 7 viruses, and then were detected by use of the multiplex-PCR with mixed five primers including two biotinylated forward primers of HHV-f and EV-f and three reverse primers of HHV-r, EBV-r and EV-r [Table 1]. The PCR products were then subjected to 2% agarose gel electrophoresis, which displayed the clear and correct bands of target fragments shown in Fig. 2A and Table 1. During this process, the negative CSF without viral controls was used as the negative control.

After amplification, the PCR products were subsequently hybridized with the viral microarray, showing the satisfying results with neither cross-reaction nor misidentification for HSV1, HSV2, EBV, CMV and EV71, but inperceptible cross-reaction between CA16 and CB5 shown in Fig. 2B.

### Sensitivity of the microarray assay

**Fig 3:**
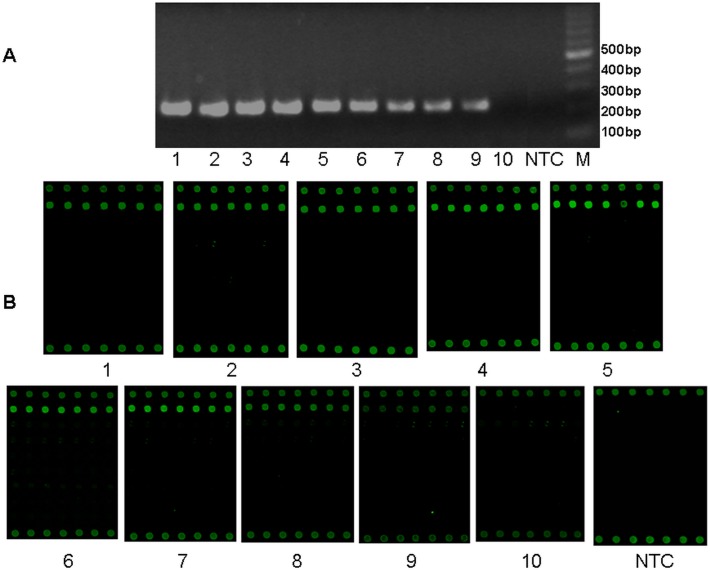
Detection sensitivity of multiplex-PCR-microarray for HHVs. The detection sensitivity was estimated for HHVs (HSV1) by multiplex RT-PCR and microarray. A. Multiplex RT-PCR result from serial diluted recombinant HSV1 control; B. The microarray result after amplification and hybridization. M: 1 Kb DNA ladder; NTC: no template control. Numbers 1–10: Serial 10-fold dilutions of recombinant HSV1 control. 1: 6×10^8^copies/μl; 2: 6×10^7^copies/μl; 3: 6×10^6^copies/μl; 4: 6×10^5^copies/μl; 5: 6×10^4^copies/μl; 6: 6×10^3^copies/μl; 7: 6×10^2^copies/μl; 8: 6×10^1^copie/μl; 9: 6×10^0^copies/μl; 10: 6×10^–1^copies/μl.

**Fig 4:**
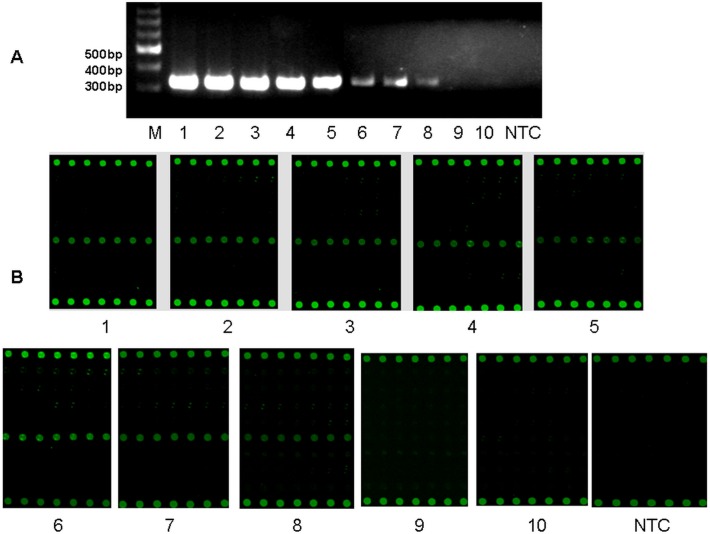
Detection sensitivity of multiplex-PCR-microarray for EVs. The detection sensitivity of the assay was estimated for EVs (EV71) by multiplex RT-PCR and microarray. A. Multiplex RT-PCR result from serial diluted recombinant EV71 control; B. The microarray result after amplification and hybridization. M: 1 Kb DNA ladder; NTC: no template control. Numbers 1–10: Serial 10-fold dilutions of recombinant EV71 control. 1: 6×10^8^copies/μl; 2: 6×10^7^copies/μl; 3: 6×10^6^copies/μl; 4: 6×10^5^copies/μl; 5: 6×10^4^copies/μl; 6: 6×10^3^copies/μl; 7: 6×10^2^copies/μl; 8: 6×10^1^copie/μl; 9: 6×10^0^copies/μl; 10: 6×10^–1^copies/μl.

Sensitivity of this multiplex RT-PCR microarray was estimated by using recombinant HSV1 and EV71 positive controls as representatives of the HHVs and EVs, respectively. The serial ten-fold dilutions from 6 × 10^8^ copies/μl to 6 × 10^–2^copies/μl were used as templates for amplification and hybridization. Fluorescence signals were scanned and analyzed. The average fluorescence intensity from all seven repetitive dots within each line was compared to the mean background intensity. The threshold of detection was determined as SNR 3.0. Any signal values lower than SNR 3.0 was regarded as negative. The detection limits were 6 copies/μl for HHVs and 60 copies/μl for EVs, respectively as shown in Figs. 3 and 4.

### Evaluation of clinical specimens

**Fig 5:**
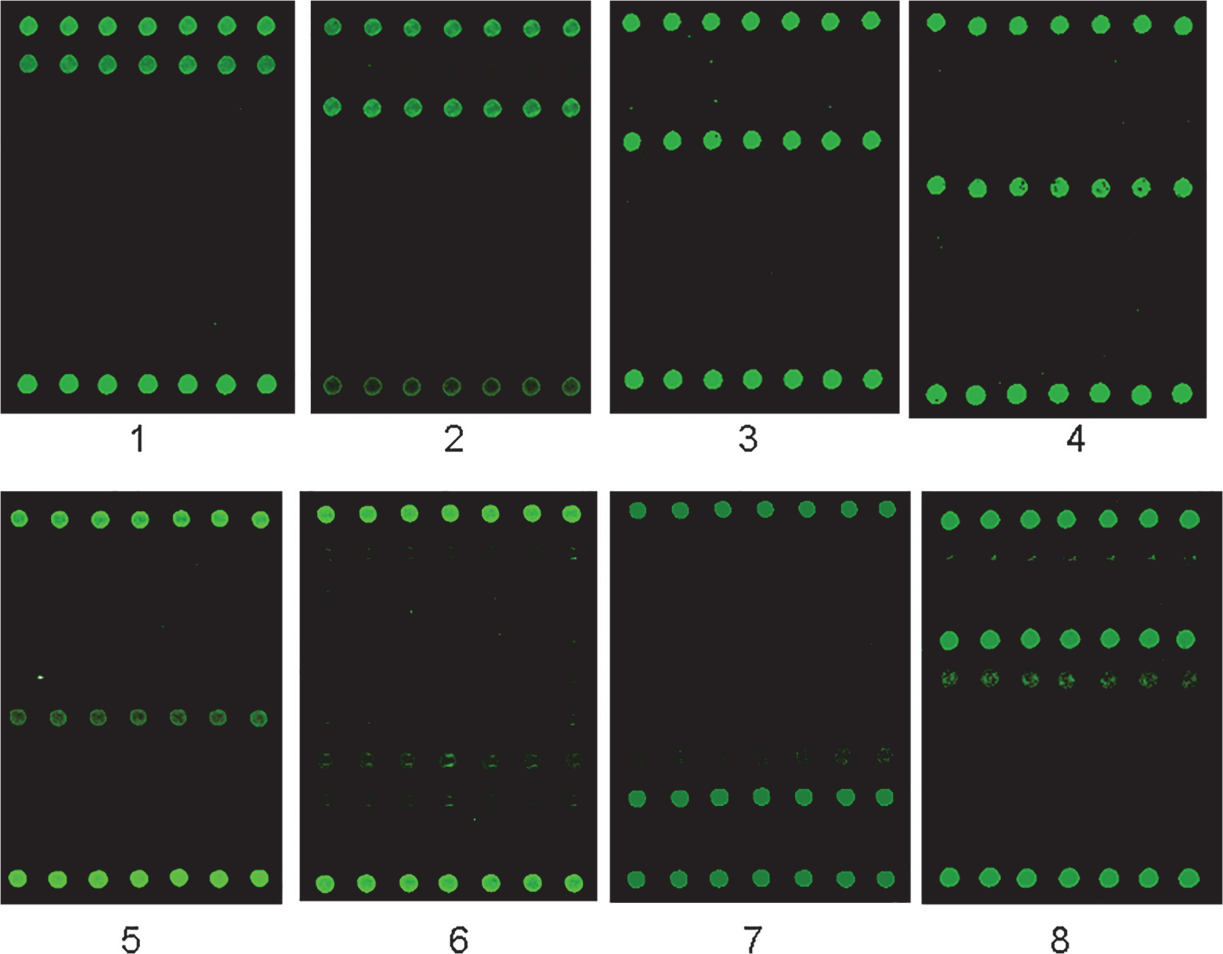
Representative detection results from clinical samples. The DNA and RNA were extracted simultaneously from clinical samples and then amplified by use of the multiplex RT-PCR followed by hybridization with the microarray. The numbers of 1–8 showed the representative results from eight clinical samples. 1: HSV1; 2: HSV2; 3: EBV; 4: CMV; 5: EV71; 6: CA16; 7: CB5; 8: co-infection of EBV and CMV.

Seven viruses were detected in two panels of clinical samples, containing 139 clinical samples with suspected systemic or neurological virus infections and 55 control samples from children of healthy or clinical symptoms uncorrelated to viral infections by use of the multiplex RT-PCR microarray. Of 139 clinical samples, 66 were positive (55 blood and 11 CSF samples) for the 7 viruses, including 13 positive for HSV1, 7 for HSV2, 5 for EBV, 24 for CMV, 1 for both EBV and CMV, 11 for EV71, 3 for CA16 and 2 for CB5 (Fig. 5, Table 3, array data from S1_data to S96_data in S1 File). Compared with the results of TaqMan qPCR/qRT-PCR which identified 71 positive (60 blood and 11 CSF samples) from the 139 samples and none from the 55 samples, the diagnosis sensitivity, specificity and Cohen's Kappa of the microarray for detection of each virus were calculated. The diagnosis sensitivity, specificity and Cohen's Kappa of the microarray was 100%, 100% and 1.0 for HSV1, HSV2, EV71 and CB5; 80%, 96.27% and 0.63 for EBV; 95.83%, 98.26% and 0.91 for CMV; 71.43%, 98.52% and 0.74 for CA16. There were no statistic significance between the two assays (*p*>0.05) analyzed using Chi square test for all viruses. The Cohen's Kappa values showed almost perfect agreement in term of HSV1, HSV2, CMV, EV71 and CB5, but lower Kappa values were noticed for EBV (0.63) and CA16 (0.74) indicating moderate to substantial agreement. The detection specificities for all viruses were high from 96.27 to 100% but the sensitivities were various from 71.43% for CA16, 80% for EBV, 95.83% for CMV to 100% for HSV1, HSV2, EV71 and CB5 in comparison with qPCR/qRT-PCR. Among 55 samples, no positive was found for these viruses by microarray and qPCR/qRT-PCR.

**Table 3:** Comparison of microarray and qPCR/qRT-PCR for detection of viruses.

Virus	qPCR/qRT-PCR/sequencing		microarray		Sensitivity %	Specificity %	Cohen's Kappa
	Positive (%)	Negative (%)	Positive (%)	Negative (%)			
**HSV1**	13 (9.35)	126(90.65)	13 (9.35)	126 (90.65)	100	100	1
**HSV2**	7 (5.04)	132 (94.96)	7 (5.04)	132 (94.96)	100	100	1
**EBV**	9 (6.47)	130 (93.53)	6 (4.32)	133 (95.68)	80	96.27	0.63
**CMV**	24(17.27)	115 (82.73)	25(17.99)	114 (82.01)	95.83	98.26	0.91
**EV71**	11(7.91)	128(92.09)	11(7.91)	128(92.09)	100	100	1
**CA16**	5(3.60)	134(96.40)	3(2.16)	136(97.94)	71.43	98.52	0.74
**CB5**	2(1.44)	137(98.56)	2(1.44)	137(98.56)	100	100	1

Two samples showed CB5 positive in microarray assay were performed conventional RT-PCR followed by sequencing because there was no available CB5 TaqMan qRT-PCR kit and universal EVs positive, EV71 and CA16 negative by using the three types of EVs qRT-PCR kits. The results of sequencing showed the homologous sequences between both samples and CB5 isolated strain VR-1036 were 97% and 95%, separately, which were consistent with the results in microarray assay. There were two CA16 positive samples in qRT-PCR showing negative in microarray. Therefore we did sequencing for them and discovered their target sequences were the CA16 variants which showed only 14 base pairs identical with the CA16 probe sequence. We further analyzed the differences of both assays for EBV. Three EBV positive samples identified by qPCR were reported to be negative by microarray. We traced their the qPCR detection details which indicated EBV copies were all slightly higher than threshold in qPCR assay but a little lower than NRS 3.0 in microarray. One sample of EBV and CMV positive was also identified in the microarray demonstrating its capability to detect co-infection of different viruses.

## Discussion

Viruses are the major cause of CNS infections, ahead of bacteria, parasites and fungal agents [22]. The use of real time PCR (qPCR) has revolutionized clinical viral diagnostic approaches [4,23]. At present, increasing numbers of HHVs and EVs have been reported to be causative pathogens in CNS infections, however, current detection method for HHVs and EVs has still to be carried out at the separated multiple steps: first, to extract DNA and RNA from HHVs and EVs with proteinase K/phenol-chloroform-isaamyl alcohol and Trizol/chloroform-isopropanol,; second, to run PCR for DNA materials and RT-PCR for RNA materials; third, to identify specific viruses with different hybridizations or sequencing. The total elapsed time is more than one day. More importantly, the amount of sample required in the current method is at least 400 μl. So a comprehensive high throughput identification method is warranted. In this study, we described a rapid and specific detection method for two genera of viruses of HHVs and EVs from the same sample. The viral microarray was restricted to four HHVs (HSV1, HSV2, EBV and CMV) and three EVs (EV71, CA16 and CB5). The total process time was only 5.5 hours, including simultaneous extraction of HHVs DNA and EVs RNA with a GuSCN buffer and subsequently simultaneous amplification with the biotinylated asymmetry multiplex-RT-PCR followed by hybridization with the microarray. The required sample size was only 100 μl which is especially necessary for infants and young children as the samples are very precious and would be insufficient if using the current methods.

In multiplex PCR, primer compatibility problem often occurs when different pairs of primers are mixed together in one single reaction, and the variable suppression effects on some of primer pairs may cause false-negative results due to primers secondary structures and /or different G+C contents[12]. To minimize the potential suppression effects, we selected conservative genes to design the universal primers that there would be as less primer sets as possible. After studying the different genes from various HHVs, highly conservative DNA polymerase gene was determined as the target gene to design primers and probes [15–19]. In regard to EVs, high conservation of the 5’-untranslated region (5’-UTR) has been reported and applied to develop microarray for detection of EVs [15,24–26]. We analyzed the 5’-UTR sequences of different EVs from GenBank database, one pair of primers were designed to amplify the majority of enteroviruses. In total, four HHVs and three EVs were determined as the target viruses in the study. The probes were designed based on the variable regions between forward and reverse primers.

Fluorophore labels used commonly for microarray analysis are Cyanine 3 (Cy3) and Cyanine 5 (Cy5) which exhibit efficient quantum yields, moderate photostability, unique excitation and emission spectra but with lower sensitivity in comparison with TaqMan qPCR [12,17,27]. To enhance detection sensitivity of this viral microarray, the biotin-streptavidin—Cy3 signal amplification system was used and an asymmetric multiplex RT-PCR was established by adding biotin labeled forward primers to reverse primers of HHV-r and EV-r in a proportion of 9 to 1 and even much lower for EBV-r due to its higher amplification efficiency. The detection limits of this viral microarray were 6 copies for HHVs and 60 copies for EVs. In this study, the sensitivity was performed with recombinant viral controls, which could be lower in infected cells or clinical samples because material extracted from cells or clinical samples could contain additional factors such as cellular DNA, RNA and trace protein that would negatively impact the efficiency of PCR and therefore reduce sensitivity. This asymmetry multiplex RT-PCR microarray was optimized by using a small numbers of confirmed positive clinical samples and will be further applied to more clinical samples in order to improve the application value for clinical diagnosis.

The asymmetry multiplex RT-PCR microarray provides an innovation technique of simultaneous extraction, amplification and hybridization of viruses with simplicity and specificity. It only takes approximately five and half hours from extraction of viral genome to RT-PCR amplification and microarray hybridization with only 100 μl of samples, demonstrating its suitability for the detection of both human herpesviruses and enteroviruses from one single sample including CSF. More importantly, this array is flexible and easy to enlarge and modify to detect various human herpesviruses and enteroviruses for routine application in clinical laboratory.

## Supporting Information

S1 File Array data from 66 positive and 30 negative samples can be found with this article, which labeled S1_Data, S2_Data, S3_Data, …… S96_Data, individually. (ZIP)
